# IL-1 Receptor-Associated Kinase Signaling and Its Role in Inflammation, Cancer Progression, and Therapy Resistance

**DOI:** 10.3389/fimmu.2014.00553

**Published:** 2014-11-17

**Authors:** Ajay Jain, Sabina Kaczanowska, Eduardo Davila

**Affiliations:** ^1^Division of Hepatobiliary and Pancreatic Surgery, Department of Surgery, State University of New York Upstate Medical University, Albany, NY, USA; ^2^Department of Microbiology and Immunology, University of Maryland School of Medicine, Baltimore, MD, USA; ^3^Greenebaum Cancer Center, Baltimore, MD, USA

**Keywords:** IRAK-4, cancer, toll-like receptors, therapeutics, inflammation

## Abstract

Chronic inflammation has long been associated with the development of cancer. Among the various signaling pathways within cancer cells that can incite the expression of inflammatory molecules are those that activate IL-1 receptor-associated kinases (IRAK). The IRAK family is comprised of four family members, IRAK-1, IRAK-2, IRAK-3 (also known as IRAK-M), and IRAK-4, which play important roles in both positively and negatively regulating the expression of inflammatory molecules. The wide array of inflammatory molecules that are expressed in response to IRAK signaling within the tumor microenvironment regulate the production of factors which promote tumor growth, metastasis, immune suppression, and chemotherapy resistance. Based on published reports we propose that dysregulated activation of the IRAK signaling pathway in cancer cells contributes to disease progression by creating a highly inflammatory tumor environment. In this article, we present both theoretical arguments and reference experimental data in support of this hypothesis.

## Introduction

Interleukin-1 receptor-associated kinases (IRAK) play a central role in inflammatory responses by regulating the expression of various inflammatory genes in immune cells. These signals are critical for elimination of viruses, bacteria, and cancer cells, as well as for wound healing. Inflammation plays contradictory roles in tumor development, exhibiting both the potential to promote anti-tumor immune responses and also paradoxically to support tumor growth and metastases. What role the expression of IRAK family members in cancer cells plays in tumorigenesis and cancer progression remains relatively unknown and is the focus of this review. We also describe how these proteins may be novel therapeutic targets that can be inhibited in order to sensitize cancer cells to cytotoxic therapies.

**Figure 1:**
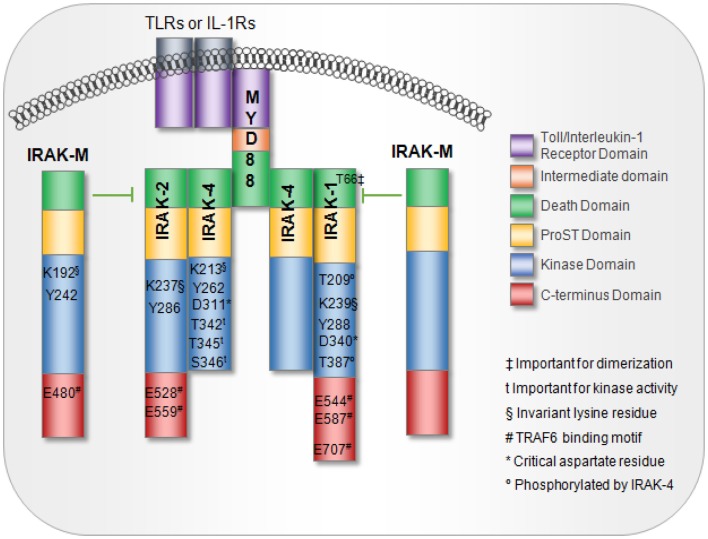
**IL-1 receptor-associated kinase family members and domains**. MyD88 interaction with TLRs or IL-1R receptors is mediated via interactions between the toll-interleukin receptor (TIR) domains. MyD88 recruitment to TLRs or IL-1R induces IRAK proteins to associate with MyD88 through death domains. IRAK-M blocks IRAK dissociation from the receptor complex, thus, acting as a negative regulator of downstream signaling. Key residues important for activation are noted.

The IRAK family is composed of IRAK-1, -2, and -4, which are expressed in a variety of human immune cell types and IRAK-M whose expression is largely limited to monocytes and macrophages (1), Figure 1. Greater details regarding the structures of the IRAK family proteins were extensively described in a recent review by Flannery and Bowie (1). All four IRAK family proteins contain an N-terminal death domain (DD), a ProST domain, and a centrally located kinase domain (1). With the exception of IRAK-4, all IRAK family members also contain a C-terminal domain. The DD serves as a platform that allows protein–protein interaction with other DD-containing proteins, the most important of which is the adaptor protein myeloid differentiation factor 88 (MyD88) (1, 2).

The proST domain, which contains serine, proline, and threonine residues, is important for regulating some of the IRAK family proteins. For example, in IRAK-1, auto-phosphorylation occurs several times in the ProST domain, which is located between the N-terminal DD and the kinase domain. Phosphorylation at multiple sites allows IRAK to dissociate from MyD88 while maintaining interactions with downstream proteins such as TNF receptor-associated factor 6 (TRAF-6) to initiate signaling (1, 3). Furthermore, all IRAK proteins contain an invariant lysine in subdomain II of the kinase domain. This invariant lysine is essential for ATP binding and catalytic function, and disruption of this lysine abrogates kinase activity (1, 4). IRAKs also contain a tyrosine “gatekeeper” residue (Tyr^262^) that alters the conformation of the IRAK protein, allowing it to maintain an active orientation. The term “gatekeeper” arises from its role in blocking a hydrophilic pocket located behind the ATP-binding site where small-molecule ATP competitive inhibitors bind and impair function (5). In a database search of over 400 kinases, this Tyr^262^ residue was seen exclusively on IRAK family members (5). Finally, IRAK proteins can initiate downstream activation of NF-κB and JNK through engagement and activation of TRAF-6 (1, 6). Interaction with TRAF-6 occurs through Pro-X-Glu-X-X-(Ar/Ac) motifs located in the C-terminal region of IRAK1-3 (1, 6).

## IRAK Activation

**Figure 2:**
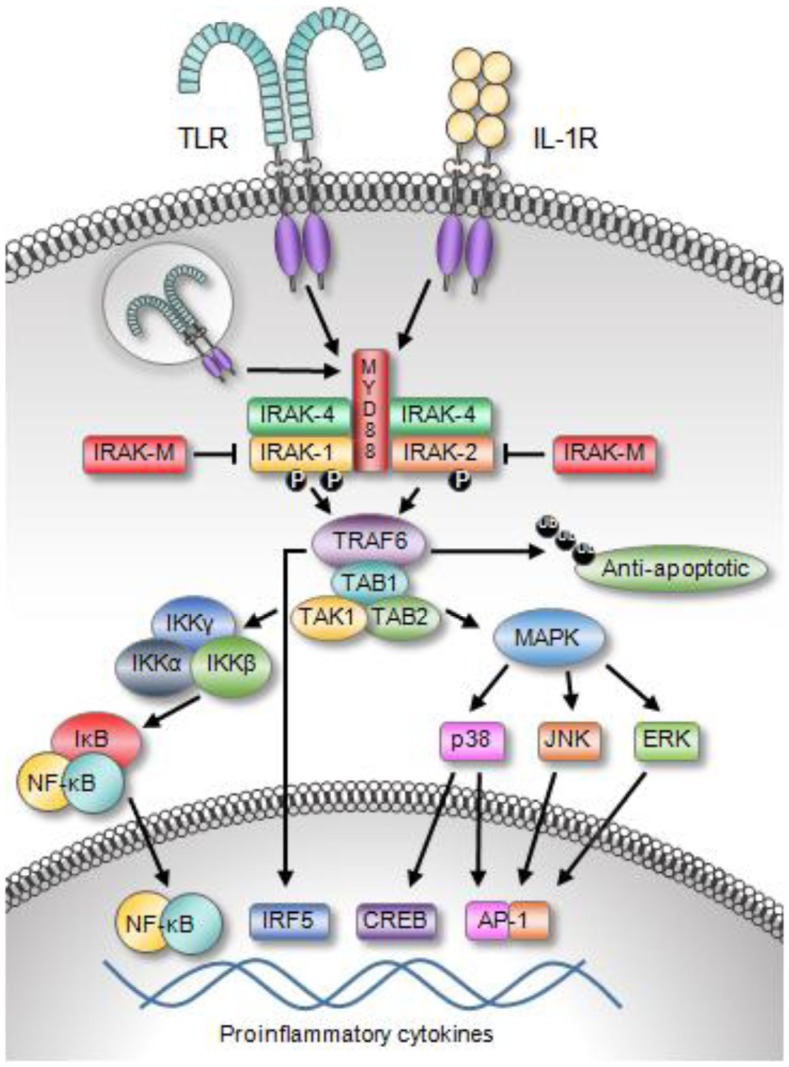
**Toll-like receptor and IL-1R family members activate IRAK signaling**. The engagement of TLRs or the IL-1R recruits MyD88 and IRAK family proteins to the receptor complex. Upon activation, IRAK members associate with TRAF6, which leads to the activation of a variety of transcription factors, including NF-κB, IRF5, AP-1, and CREB. The activation of these transcription factors results in the expression of a broad array of inflammatory molecules and apoptosis-related proteins. Moreover, TRAF6 can alter protein stability though its ability to polyubiquitinated various proteins including anti-apoptotic proteins.

IL-1 receptor-associated kinase signaling can be initiated from Toll-like receptors (TLRs) or from the interleukin-1 family receptors (IL-1R), Figure 2 (7, 8). Thirteen TLRs have been identified in human beings. TLRs recognize conserved pathogen-associated molecular patterns (PAMPs) expressed on a variety of microbes including bacteria, fungus, yeast, and viruses. Some TLRs can also be stimulated by endogenous danger signals released from stressed or dying cells such as HMBG-1 and A100 (9, 10). A wide variety of cancers have been shown to express functional TLRs. A detailed review regarding the expression of TLRs and the consequence of ligating these receptors on tumor cells was recently published by Kaczanowska et al. (11). The IL-1Rs bind pro-inflammatory cytokines in the IL-1 family, the most well-known of which are IL-1α, IL-1β, and IL-18. The signaling cascade is initiated by the adaptor MyD88 binding to the toll/interleukin-1 receptor (TIR) domain, which is shared by these receptors. MyD88 oligomerizes and recruits IRAK-4 via the DD. IRAK multimerization is dependent on DD interactions, which in turn result in kinase activation and propagation of the downstream signal.

Of the four IRAK proteins, IRAK-1 and IRAK-4 are active serine/threonine kinases (12). IRAK-4, the most recent IRAK family protein to be discovered, is the most proximal IRAK family protein in the TIR-mediated signaling pathway and directly downstream of MyD88 (8, 13, 14). IRAK-4 and IRAK-1 are able to associate with each other upon engaging MyD88 through their DD. IRAK-4 is thought to phosphorylate IRAK-1, which allows IRAK-1 to initiate an auto-phosphorylation cascade occurring in three sequential steps (15). IRAK-1 is first phosphorylated at Thr^209^, which causes a conformational change in the protein (14, 15). The second step is phosphorylation at Thr^387^. IRAK-1 does not become fully active until this residue is phosphorylated. There are data suggesting that either Thr^209^ or Thr^387^ may be sites for initial IRAK-1 phosphorylation by IRAK-4. However, this question remains unresolved as both of these residues are also sites of auto-phosphorylation. The third step is auto-phosphorylation at several residues in the proST region; this allows IRAK-1 to be released from the active receptor complex. IRAK-1 and TRAF-6 dissociate from the complex, bind TAB-1 (TAK-1 binding protein-1) followed by binding of TAK-1 (transforming growth factor-β-activated kinase) and TAB-2. IRAK-1 ubiquitination and degradation are rapidly induced. The remaining complex translocates into the cytoplasm, associates with ubiquitin ligase such as ubiquitin conjugating enzyme-13 (UBC-13) and ubiquitin conjugating enzyme E2 variant-1 (UEV-1a), leading to ubiquitination and degradation of TRAF-6. This activates TAK-1 and phosphorylation of the inhibitor of κB kinase (IKK) complex (IKKα, IKKβ, and IKKγ), as well as mitogen activated protein kinases (MAPKs). The resulting NF-κB activation regulates the transcription of pro-inflammatory genes. IRAK-1 activity and induction of NF-κB is also regulated by ubiquitination at Lys^134^ and Lys^180^. It is worth noting that mutant forms of IRAK containing arginine at these sites have an impaired capacity to induce NF-κB (16).

While the IRAK-1 kinase activity is also not essential for IL-1R-mediated NF-κB activation, its role as an adaptor protein that brings together MyD88, IRAK-4, and Tollip is essential for IL-1R-mediated NF-κB activation (17–19). IRAK-1 expression and activation is, of course, subjected to regulation. In addition to inducing activation, auto-phosphorylation renders IRAK-1 susceptible to proteasome-mediated degradation (17, 19). Regulation may also occur at a transcriptional level (19). For example, a human IRAK-1b splice variant that lacks kinase activity is resistant to proteasome-mediated degradation, and an IRAK-1c splice variant with a truncated sequence at the C-terminal end of the kinase domain functions as a *negative* regulator of TLR and IL-1R signaling (17, 20, 21).

IRAK-2 was initially thought to be a “pseudokinase” because a critical aspartate residue in the catalytic domain is replaced with asparagine and unlike IRAK-1 and IRAK-4, IRAK-2 cannot autophosphorylate (22–25). However, IRAK-2 possesses catalytic activity and has been implicated in maintenance of pro-inflammatory cytokine release induced by TLR4 and TLR9 engagement (24). Wesche et al. demonstrated that wild-type IRAK-2 can be phosphorylated when co-cultured with IRAK-1. Although it is not as good a substrate as wild-type IRAK-3, it can replace IRAK-1 when IRAK-1 is knocked down (25). However, a mutant IRAK-2 containing a substitution (K237A) in its ATP-binding pocket is not able to be phosphorylated (23, 25). Kawagoe et al. confirmed that IRAK-4, and not IRAK-1, phosphorylates IRAK-2, resulting in activation which essential for IRAK-2 kinase and effector function.

Similar to the other IRAK proteins, IRAK-3 (a.k.a. IRAK-M) can form complexes with MyD88 and TRAF-6, Like IRAK-2, it is considered to be a pseudokinase with very limited capacity for auto-phosphorylation, but with the potential to become activated by other IRAK proteins and serve as a functional kinase. In contrast to other IRAK proteins, IRAK-M is thought to function as a negative regulator that prevents the dissociation of IRAK-1 and IRAK-2 from the receptor complex, inhibiting their interaction with TRAF-6 and interrupting the downstream inflammatory cascade (26, 27).

More recent data show that IRAK-M may promote anti-inflammatory effects through a paradoxical “second wave” of NF-κB activation. In this model, IRAK-M interacts with the MyD88/IRAK-4 complex to form an IRAK-M Myddosome. Upon ligation of the IL-1R, the IRAK-M Myddosome can induce a second wave of NF-κB activation and is dependent on MEKK3 signaling (26). However, this secondary NF-κB activation is believed to decrease overall inflammation by inducing the expression of several inhibitory molecules such as SOCS1, SHIP1, A20, and IκBα (20). IRAK-M can also interact with IRAK-2 in order to inhibit mRNA transcription of inflammatory cytokines and chemokines.

## Roles of the Different IRAK Family Proteins in Cancer

### IRAK-1

**Figure 3:**
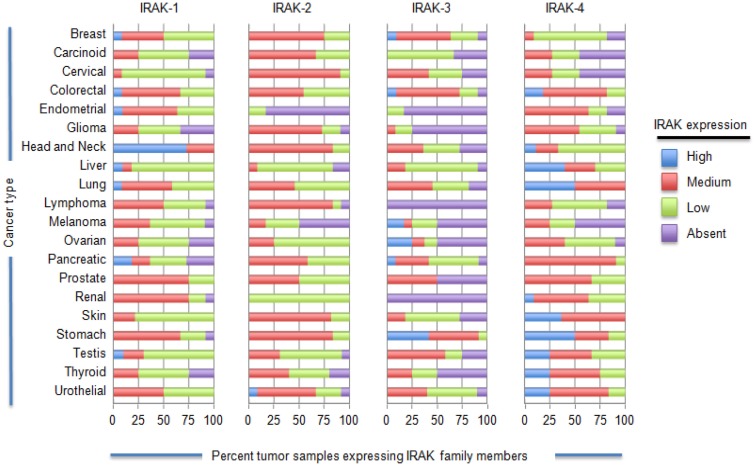
**IL-1 receptor-associated kinase expression on a variety of tumor cells**. ProteinAtlas (www.proteinatlas.org/cancer) was used to determine the IRAK protein expression patterns in the various human cancer specimens shown. Protein expression profiles are based on immunohistochemistry from human specimens. The number of samples for each specimen are as follows: breast, 12; carcinoid, 4; cervical cancer, 11; colorectal cancer, 12; endometrial cancer, 11; glioma, 12; head and neck, 4; liver, 11; lung cancer 11; lymphoma, 12; melanoma, 12; ovarian, 12; pancreatic, 12; prostate, 11; renal, 12; skin, 11; stomach, 12; testis, 12; thyroid, 4; urothelial, 12.

There is an increasing body of data to suggest that IRAK-1 signaling may be important to the development and progression of cancer. *Helicobacter pylori*, bacteria strongly associated with gastric inflammation and the development of gastric cancer has been shown to cause upregulation of TLR2 and TLR5 expression in various cell types and subsequent engagement of these receptors increases IRAK-1 phosphorylation and NF-κB activation (1). Importantly, gastric carcinogenesis was recently reported to be associated with increased TLR expression and reduced expression of the TLR inhibitors Tollip and PPAR (2). As another example, an evaluation of over 300 tumor samples from non-squamous cell lung cancer (NSCLC) patients showed that tumor tissue had significantly increased cytosolic IRAK-1 expression and decreased nuclear expression relative to adjacent normal tissue (3). Our group has also found IRAK-1 and/or IRAK-4 to localize to the nucleus of melanoma cells, but not melanocytes (Geng, unpublished data). IRAK’s role in the nucleus and how this contributes to tumor progression has not been defined. In order to gain a better sense of the expression levels of each IRAK family member in various cancer types, we analyzed immunohistochemistry data using the online data base ProteinAtlas (http://www.proteinatlas.org/), Figure 3. These data highlight the heterogeneity of different IRAK family members in different cancer types. Of all the IRAK family members, IRAK-4 was the most frequently expressed (at the medium to high range) and found on the highest percentage of tumor samples. IRAK-1 was the next most frequently expressed with appreciable levels (medium to high) in all tumor samples analyzed. IRAK-2 and IRAK-3 were the least detected IRAK family members, respectively. Despite the high-expression levels of IRAK-1 and IRAK-4, it is important to note that the level of activation (phosphorylation) was not examined but plays an important role in IRAK signaling.

Additional evidence indicating the importance if IRAK-1 in cancer came from studies of microRNAs (miRNAs) (4). miRNAs are small non-coding RNA sequences that play critical roles in regulating cellular mRNA stability, protein expression, proliferation, apoptosis, and cancer metastasis (5, 6). It has been shown that expression of a specific miRNA (miR-146a) is frequently diminished in metastatic prostate cancers. Intriguingly, upregulation of miR-146a and miR-146b in metastatic breast cancer cell lines has been shown to downregulate TRAF-6 and IRAK-1 expression and subsequently reduce NF-κB expression (5, 28, 29). Moreover, inhibiting miR-146a expression also reduced cancer cell invasiveness of pancreatic and colon cancer cell lines. Panc-1 and Colo-1 pancreas and colon cancer cell lines, respectively, also have lower miR-146 expression in comparison to non-malignant pancreas cells, and induction of miRNA in these cancers lines decreases their invasiveness. This phenotypic change is also accompanied by down-regulation of EGFR and metastasis-associated protein 2 (MTA-2) (5).

IRAK-1 may be particularly relevant to the pathogenesis of melanoma. The use of rapid subtraction hybridization analysis was used to identify IRAK-1 as one of eight genes that are differentially expressed in metastatic cells compared to parental human melanoma cell lines, with IRAK-1 expression being upregulated in the metastatic variants (5, 30). Srivastava et al. reported that a large percentage of established human melanoma cell lines exhibit constitutive expression of phosphorylated forms of IRAK-1 and IRAK-4 (31). Patient-derived melanoma tumor samples also exhibited increased expression of phosphorylated IRAK-4 although there did not appear to be a correlation between p-IRAK levels and melanoma stage. Inhibition of IRAK-1 and IRAK-4, using pharmacological inhibitor or siRNA, sensitized melanoma tumors expressing phosphorylated forms of these IRAKs to cytotoxic chemotherapies *in vivo*, raising the possibility that IRAK family proteins may be potential therapeutic targets in cancer. In agreement with these studies, recent data indicate that inhibiting IRAK-1,-4 signaling in a variety of leukemias including Waldenstrom macroglobulinemia, diffuse large B-cell lymphoma, myelodysplasia, and acute myeloid leukemia substantially impaired proliferation *in vitro* and *in vivo*, and treatment with IRAK inhibitors prolonged mouse survival (32, 33). We recently found that IRAK-4 signaling in T cell acute lymphoblastic leukemia (T-ALL) is critical for their ability to proliferate but did not induce cell death (Li, unpublished data). In order to determine whether IRAK inhibitors could enhance the cytotoxic effects of chemotherapeutic agents, we screened nearly 500 FDA-approved drugs for their ability to kill T-ALL cells when combined with IRAK inhibitors. We identified three classes of drugs that worked synergistically with IRAK inhibitors and, in some cases, restored sensitivity of chemoresistant samples. Whether a similar effect will be observed in other cancer types merits further investigation. This is especially true given that many cancers exhibit increased protein levels of IRAK-1 and IRAK-4 and are resistant to chemotherapy (Figure 3).

Finally, IRAK-1 activation may also be important for cross talk between cancer cells and other cell populations present in the tumor microenvironment. IL-1β release by lingual squamous cell carcinomas causes upregulation of the IL-1R and increased levels of p-IRAK-1 in cancer associated fibroblasts. This results in nuclear translocation of NF-κB and induction of genes important for tumor progression including IL-6, Cox-2, BDNF, and IRF-1 (34).

### IRAK-2

In terms of signaling and function, there is some redundancy between IRAK-2 and IRAK-1. Using single and double IRAK knockout mice, Kawagoe and colleagues confirmed that both IRAK1 and IRAK2 have common functionality in the early phase of TLR signaling (23). IRAK2 kinase activity, however, was longer sustained than that of IRAK-1, and IRAK-2 was critical in late-phase TLR responses. This raises the possibility that IRAK-2 may be relevant to chronic inflammatory responses often associated with cancer. Whether downstream signaling differs between IRAK-1 and IRAK-2 remain to be determined. Recent studies by Cui and colleagues suggest that a stress-induced NF-κB-activated, miRNA-146a-mediated down-regulation of IRAK-1 coupled to an NF-κB-driven upregulation of IRAK-2 supports a self-perpetuating inflammatory signaling loop (35).

The role of IRAK-2 as a regulator of TLR signaling may be more complex than originally thought. IRAK-2 is known to induce NF-κB activation through TLR3, TLR4, and TLR8 (14). Of note, IRAK-2 is the only member of the family thought to mediate signaling through TLR3. Interestingly, IRAK-2 has recently been shown to have a dual function (immunosuppressive and immunostimulatory) in TLR9 related signaling and inflammatory responses. Wan and colleagues demonstrated that IRAK-2 suppresses TLR9 signaling in the early post-stimulation phase, raising the activation threshold for TLR9-induced inflammatory response and potentially preventing autoimmunity (36). However, if the higher activation threshold is successfully triggered through a strong stimulus, IRAK-2 mediates a positive feedback loop allowing for sustained release of pro-inflammatory cytokines. It is conceivable that loss of negative regulatory function could allow sustained IRAK-2 activation and inflammation, thus, promoting carcinogenesis. Importantly, whereas TLR9 was previously thought to be expressed only on immune cells, it has been shown that it also expressed on a number of different cancers (oral, prostate, breast, lung, Burkitt lymphoma), and signaling through TLR9 promotes proliferation and/or cell survival (37–44).

### IRAK-3 (a.k.a. IRAK-M)

Unlike other IRAK family members that are widely expressed on a variety of cell types, IRAK-M is thought to chiefly reside in monocyte and macrophage populations. As mentioned previously, IRAK-M activation generally acts as a negative regulator of NF-κB activation in TLR and IL-1R signaling (45). Also, even though IRAK-M induces a paradoxical “second wave” of MEKK3 dependent NF-κB activation, the overall effect of IRAK-M favors immunosuppression (26).

IRAK-M is a negative regulator of IRAK-4/IRAK-1 and IRAK-4/IRAK-2 and thus serves to inhibit the expression of a variety of inflammatory molecules induced by IRAK-4. Our working hypothesis is that in cancers with reduced levels of IRAK-M but elevated levels of IRAK-1, -2, and/or -4 will show increased IRAK-4 signaling and consequently elevated levels of inflammatory molecules. In addition to augmenting the amounts of inflammatory factors, the lack of IRAK-M might further sustain IRAK-4 signaling and perpetuate a chronically inflamed tumor environment; chronic inflammation is a hallmark of tumorigenesis and tumor progression (46). That IRAK-3 expression levels are reduced in some cancer types is further highlighted in Figure 3 and supports our hypothesis.

Even though it is an anti-inflammatory mediator, IRAK-M may still play an important role in tumorigenesis through modulation of the activity of tumor-associated macrophages (TAMs). It is generally thought that there are two types of macrophages associated with cancer (47). These include classically activated (M1) macrophages that secrete pro-inflammatory cytokines and present antigens to cytotoxic immune effector cells, and alternatively activated (M2) macrophages with impaired Th1-like cytokine release (and one favoring Th2 cytokines) and decreased capacity to activate T cells. The M1 type is thought to play a more prominent role in the early stages of carcinogenesis through NF-κB activation and chronic inflammation to initiate carcinogenesis. As cancers become more established, M1 macrophages may become “re-educated” to take on a M2 phenotype. M2 macrophages can secrete tumor growth factors, promote angiogenesis and invasiveness through remodeling of the tumor matrix, and induce immune tolerance. The term “tumor-associated macrophage” or TAM is typically associated with the M2 phenotype. Indeed, macrophage re-education may be a critical aspect of cancer pathogenesis, and IRAK-M may play a significant role in this process.

IRAK-M may promote cancer progression through modulation of macrophage activity. IRAK-M is known to be an important negative regulator in macrophages in models of inflammation. For example, in mouse models of myocardial infarction, upregulation of IRAK-M in cardiac macrophages reduces myocardial inflammation and prevents adverse cardiac remodeling (45). Naïve monocytes and macrophages exposed to tumor cell lines exhibited decreased expression of TNFα, IL-12p40, and IRAK-1 (48, 49). Moreover, these characteristics, as well as the ability to present antigens, were diminished with prolonged exposure to tumor cells as the macrophages take on an M2 phenotype. A hallmark feature of this transition is the rapid upregulation of IRAK-M in macrophages upon exposure to tumor cells (48, 49). *In vivo* mouse studies using Lewis lung cancer (LLC) cell lines have shown that tumor infiltrating macrophages have higher IRAK-M expression and impaired ability to secrete IL-12, TNFα, and IFN-γ compared to peritoneal macrophages isolated from the same mouse (50). Interestingly, the ability of TAMs to secrete TNFα could be restored by knocking down IRAK-M expression using siRNA (48). These data indicate that IRAK-M upregulation can be induced by surface-associated or soluble factors from tumor cells to promote tumor growth and immune evasion. Proposed mechanisms include the engagement of hylauronan (a tumor cell surface glycosaminoglycan) to monocyte-expressed CD44 or secretion of TGF-β. Furthermore, monocytes isolated from patients with chronic myleogeneous show upregulation of IRAK-M mRNA, monocytes from chronic lymphocytic leukemia patients (in whom IRAK-M expression was not evaluated) showed impaired ability to secrete cytokines and present antigen. Analysis of a cohort of 439 lung cancer patients showed that the level of IRAK-M expression on tumor cells was a significant and independent predictor of mortality. In contrast, these data suggest that IRAK-M is a critical mediator of cross talk that occurs between tumor cells and macrophages to allow a more favorable tumor microenvironment and facilitate cancer progression (48, 49).

### IRAK-4

IRAK-4, the most recently identified member of the family, is considered the “master IRAK” because it is required for all MyD88-dependent NF-κB activation and for inducing IFNα expression through TLR 7, 8, and 9 (51). Loss of IRAK-4 renders mice completely resistant to LPS-induced shock, and deficiencies in human beings have been associated with increased susceptibility to encapsulated bacterial infections (especially pneumococcal) (52, 53). Data regarding the specific role of IRAK-4 in cancer have not been fully investigated, and its potential role in cancer progression is just now beginning to emerge. As previously discussed (in the Section IRAK-1) some melanomas constitutively express active, phosphorylated forms of IRAK-1 and IRAK-4. Inhibiting IRAK-4 rather than IRAK-1 using shRNA was more effective at sensitizing melanoma tumors and T-ALL cells to chemotherapies. It is still unclear, however, whether this is a direct phenomenon or whether upstream signaling events drive phosphorylation. As IRAK-4 is a lynchpin for MyD88-mediated pro-inflammatory signaling, it can promote carcinogenesis regardless of whether it is directly mutated or not. For example, a subset (29%) of activated B-cell type diffuse large B-cell lymphomas (ABC DLBCL) with a very aggressive phenotype were recently found to carry an oncogenic MyD88 mutation (L265P) that promotes survival. This mutation allowed spontaneous formation of a stable complex between MyD88, IRAK-4, and a phosphorylated form of IRAK-1. However, knockdown of IRAK-1 kinase activity was not required for survival of ABC DLBCLs, while IRAK-4 kinase activity was essential (54). To date, no group has reported any mutations in any of the IRAK family members specifically in cancer but this subject merits further investigation considering recent data uncovering an important role for dysregulated IRAK signaling via MyD88 mutations.

## IRAK Family Protein Inhibitors as Novel Cancer Therapeutics

### Small-molecule inhibitors

Given the strong data indicating that IRAK family proteins are critical mediators of inflammation, there has been considerable interest in developing targeted agents to treat autoimmune and inflammatory diseases. As we previously addressed, IRAK inhibitors (especially IRAK-1 and -4) may also have therapeutic applications in cancer. Several classes of IRAK-4 inhibitors have been developed, including amino-benzimidazole, thiazole, or pyridine amides, imidazo[1,2-*a*] pyridines, imidazo[1,2-*b*]pyridazines, and benzimidazole–indazoles (47–50, 52, 54). IRAK inhibitors may have particular utility in the treatment of Waldenstrom’s macroglobulinemia, a B-cell lymphoproliferative disorder that is critically dependent upon NF-κB activation. *However, compounds that target molecules downstream of IRAK-1 are also potential candidates*. One such compound is 5Z-7-oxozeaenol, which selectively inhibits TAK-1 and has been shown to reduce inflammation and enhance the sensitivity of breast and pancreatic cancer cells to various chemotherapeutic agents, further highlighting the central role that IRAK signaling plays in chemotherapy resistance (54–56).

### Botanical derivatives

It is possible that plant-derived compounds may also induce anti-inflammatory and anti-cancer therapeutic effects through inhibition of IRAK family members. For example, ginseng (*Panax ginseng*), which is anecdotally described to have a many health benefits including anti-inflammatory and anti-cancer properties, contains protopanaxatriol ginsenoside. This agent has been shown to inhibit IRAK-1 and IKK-β phosphorylation in LPS stimulated macrophages, as well as alleviate inflammation induced by 2,4,6-trinitrobenzene sulfonic acid-induced colitis in mice (54, 56–59). The xanthone derivative 1,3,5-trihydroxy-4-prenylxanthone (TH-4-PX) isolated from *Cudrania cochinchinensis*, a plant used as a traditional remedy for diseases in Asia, inhibits LPS/TLR-mediated release of nitrous oxide through inhibition of IRAK-1 (60). A second agent from this plant (isoalvaxanthone) has anti-neoplastic properties, as it can inhibit matrix metalloproteinase-2 expression (a factor associated with tumor invasiveness) *in vitro* in SW620 colon cancer cells. Admittedly, it is unclear if the isoalvaxanthone effects are the result of IRAK family member inhibition, as this agent did not inhibit expression of NF-κB.

## Nitrogen Bisphosphonates

There has been increasing evidence that nitrogen bisphosphonates (NPBs), a class of drugs used to treat osteoporosis, may also have potential for treating cancer. Paradoxically, NPBs are associated with inhibition of IRAK-M expression. The NBP xoledronate reduces IRAK-M levels when cultured with PBMCs from a subset of human blood donors (50%). In these individuals, the reduction in IRAK-M is associated with enhanced cytokine release after TLR stimulation or administration of IL-1 (61). Depletion of IRAK-M in dendritic cells (DCs) using siRNA has been shown to enhance DC migration to lymph nodes, augment cytokine release, and enhance antigen presentation, proliferation, and activation of antigen-specific T cells. Thus, pharmacologic inhibition of IRAK-M using NBPs may likewise improve the induction of cell-based anti-tumor immune responses. A summary of the various IRAK inhibitors is shown in Table 1.

**Table 1:** **A summary of small molecules that can inhibit IRAK family members**.

	Target
**SMALL-MOLECULE INHIBITORS**	
Amino-benzimidazole	IRAK-4
Thiazole/pyridine amides	IRAK-4
Imidazo[1,2-a] pyridines	IRAK-4 and IRAK-1
Imidazo[1,2-b]pyridazines	IRAK-4 and IRAK-1
Benzimidazole–indazoles	IRAK-4 and IRAK-1
5Z-7-Oxozeaenol	TAK1
**BOTANICAL DERIVATIVES**	
Protopanaxatriol ginsenoside	IRAK-1, IKK-β
1,3,5-Trihydroxy-4-prenylxanthone (TH-4-PH)	IRAK-1
**NITROGEN BISPHOSPHONATES**	
Xoledronate	IRAK-M

## Summary

Dysregulated IRAK signaling in tumors is beginning to emerge as an important factor in cancer initiation, tumor progression, and therapy resistance. Studies from several groups highlight the potential of IRAK family members as therapeutic targets for cancer treatment alone or when combined with other therapies. A better understanding of how IRAK signaling drives inflammation through interaction with TLR and IL-1 family members will be critical for developing targeted therapies that work synergistically with systemic chemotherapies. Furthermore, such an understanding may allow manipulation of these proteins to favor anti-tumor cytotoxicity rather than carcinogenic downstream effects.

## Conflict of Interest Statement

The authors declare that the research was conducted in the absence of any commercial or financial relationships that could be construed as a potential conflict of interest.
